# Effect of Damaged Starch and Wheat-Bran Arabinoxylans on Wheat Starch and Wheat Starch–Gluten Systems

**DOI:** 10.3390/foods13050689

**Published:** 2024-02-24

**Authors:** Andrés Gustavo Teobaldi, Gabriela Noel Barrera, Pablo Daniel Ribotta

**Affiliations:** 1 Instituto de Ciencia y Tecnología de los Alimentos Córdoba (ICYTAC-CONICET), Universidad Nacional de Córdoba, Ciudad Universitaria, Av. Filloy S/N, Córdoba CP X5000HUA, Argentina; ateobaldi@agro.unc.edu.ar (A.G.T.); gbarrera@agro.unc.edu.ar (G.N.B.); 2 Departamento de Química Industrial y Aplicada, Facultad de Ciencias Exactas, Físicas y Naturales (FCEFyN), Universidad Nacional de Córdoba (UNC), Av. Vélez Sarsfield 1611, Córdoba CP X5000HUA, Argentina

**Keywords:** damaged starch, arabinoxylans, gluten, sucrose, thermal properties

## Abstract

This study investigated the impact of damaged starch and arabinoxylans on the thermal and pasting behavior of mixtures containing starch and gluten. The mixtures containing starch, arabinoxylans, and gluten were dispersed in water and a 50% sucrose solution. When arabinoxylans were added to native starch in water, it did not modify the viscosity profiles. An increase in viscosity parameters was observed due to the addition of arabinoxylans to starch with a higher level of damage. Gluten did not influence the effects caused by arabinoxylans. In the sucrose solution, arabinoxylans caused an increase in the viscosity parameters of native starch and starch with higher damage content dispersions. Gluten caused greater viscosity increases when arabinoxylans were added. In water, the addition of arabinoxylans to native starch caused a decrease in the enthalpy of gelatinization and an increase in the onset temperature. Adding arabinoxylans to starch with a higher level of damage caused the opposite effects. In the presence of sucrose, arabinoxylans caused a decrease in the enthalpy of gelatinization. These results lay the foundations for studying the influence of damaged starch and arabinoxylans in water-rich systems characterized by the presence of substantial proportions of sucrose, such as batter formulations.

## 1. Introduction

Studying the minor components of flours and the interactions between them is an important aspect of the food industry. Starch is considered one of the inexpensive and most common polysaccharides widely distributed in nature, whether in grains, legumes, or tubers. It is generally formed by two types of polymers: linear amylose, resulting from the union of glucose units through α-(1, 4) glycosidic bonds, and the highly branched amylopectin, which contains both α-(1, 4) glycosidic and α-(1, 6) glycosidic bonds [1]. During grain grinding to obtain flour, some of the starch granules may undergo physical modifications at the surface level due to the applied forces (shear, collision, friction). This portion of physically modified starch is known as damaged starch (DS). The study of DS and its physical-chemical properties is very important in manufacturing starch-based food to predict the behavior of flour for production, since its presence can modify conditions in the processes.

Arabinoxylans (AXs) are polysaccharides present in the cell walls of various cereals. The structure consists of a linear backbone of xylose units linked by β-(1, 4) bonds with arabinose units attached to some of the xylose units. However, the structural characteristics of different AXs are complex and influenced by their source. In wheat AXs, side chains are linked by α-(1, 2) and/or α-(1, 3) bonds along the xylan backbone. The xyloses can be di-substituted, mono-substituted (the most common substitution), or not substituted at all [2].

Arabinoxylans are known to have the ability to form thick and sticky solutions, which can affect the physicochemical properties of bread, such as increased specific volume, or affect the product quality. Because of their high water absorption capacity, it is important to study the physical and chemical properties of these biopolymers to predict the behavior of certain flours when used for making specific baked products. Small additions of AXs (1% *w*/*w*) have shown to positively starch retrogradation [3]. Arabinoxylans exhibit a hydrophilic nature and this property allows them to compete with other compounds present in food matrices to capture water molecules. The hydrogen bonding capacity of AX molecules enhances the overall texture and moistness of food products. Moreover, they prevent syneresis in food products and, specifically, prevent staling in bread by retaining moisture [4]. There is scarce information on the effect of AXs in high-sucrose batter products like cakes and muffins. According to a study conducted by Moza and Gujral [5], flours that contain a high amount of AX (1.11%) can cause smaller air cells to appear in cakes. The study found that the presence of β-glucans and AX can have multiple positive effects on the batter consistency, the density of cake cells, and crumb properties. The study also concluded that these components could enhance the cake’s water-binding capacity, resulting in a moist cake crumb. Damaged starch and arabinoxylans are two minority components that have a significant impact on the formation and microstructure of baked products due to their strong interaction with water.

A previous study showed that the thermal properties and viscoelastic behavior of starch–water and starch–sucrose solution systems were affected by the level of DS [6]. However, more and deeper knowledge of the effect of wheat flour’s minor components and their interactions on the thermal and viscosity properties of starch and flour is necessary to understand the consequences on the processing of batter formulations and their products. One way to expand our knowledge is to progressively analyze the incorporation of the isolated components and their interactions.

Therefore, the objective of this study was to analyze the effect of wheat flour minor components, AX and DS, on wheat starch and wheat starch–gluten system’s thermal properties, as well as their interactions.

## 2. Materials and Methods

### 2.1. Materials

Commercial wheat starch (S) (moisture 11.0%, protein 0.6%, lipid 0.15%, and ash 0.5%, *p/p* dry basis), vital gluten (G) (moisture 10.0%, protein 75.0%, fat 1.5% and ash 1.5% *p/p* dry basis, according to the manufacturer) and wheat-bran were obtained from Molinos Juan Semino S.A. (Santa Fe, Argentina). Commercial sucrose (food grade, polarization ≥ 99.7) was purchased from a local market (Ledesma S.A.A.I., Jujuy, Argentina).

### 2.2. Extraction of Wheat Bran Arabinoxylans

The extraction method is an adaptation of two AX extraction methods [7,8]. To begin with the extraction, wheat bran (WB) was subjected to a pre-treatment to remove the starch. WB was soaked and shaken for 5 min with water at 4 °C and passed through 60 mesh. The material retained in the sieve was washed with water at 4 °C until the water drained was transparent. Washed wheat bran was dried in an oven at 60 °C overnight. After this, a process of deslipidization of WB washed and dried was conducted using hexane. The ratio of starting material to hexane was 1:10 (*w*/*v*). De-starched and deslipidized WB was milled in a hammer miller to obtain smaller WB particle sizes. The deslipidized material was then treated with a 0.15 M sodium hydroxide solution containing 0.5% (*v*/*v*) hydrogen peroxide in a 1:8 (*w*/*v*) ratio. The mixture was stirred for 1.5 h at 80 °C. After stirring, the solution was centrifuged for 30 min at 5000× *g*. The supernatant was collected and acidified with a 2 M hydrochloric acid solution until reaching about pH 4.5 to precipitate the proteins. Then, the obtained solution was centrifuged again for 30 min at 5000× *g*. Subsequently, 96% (*v*/*v*) ethanol was added to the supernatant (AX solution) until reaching the 1:2 ratio (AX solution/ethanol) to precipitate the AXs. To favor precipitation, the mixture was kept overnight in the freezer at −20 °C. Then, it was centrifuged for 5 min at 5000× *g* at 4 °C. The obtained precipitate was placed in a freezer at −40 °C and then lyophilized (dried AX extract). The arabinoxylan content was determined following the orcinol—HCl method [9].

### 2.3. Mechanical Damage of the Wheat Starch

Unmodified wheat starch (NS) (4.1% of DS) was milled in a Whisper Series Bench Top disk mill (Rocklabs, Auckland, New Zealand) to increase the DS level [6]. The DS content of the re-milled starch was 31.6% DS. Temperature was controlled during milling; it was kept under 40 °C. The DS content was determined using the AACC 76–30 A method (AACC, 2000) [10]. Each measurement was made in triplicate.

### 2.4. Samples Preparation

Starch system: Four starch samples with different DS and AX levels were prepared by mixing the solids NS, DS, and AX: (1) NS/AX4 (4.1% DS and 4% AX), (2) NS/AX8 (4.1% DS and 8% AX), (3) DS/AX4 (31.6% DS and 4% AX), and (4) DS/AX8 (31.6% DS and 8% AX).

Starch–gluten system: Four samples containing 90:10 starch/gluten proportions with different DS and AX levels were prepared by mixing the solids NS, DS, AX, and G: (1) NS/G/AX4 (4.1% DS and 4% AX), (2) NS/G/AX8 (4.1% DS and 8% AX), (3) DS/G/AX4 (31.6% DS and 4% AX), and (4) DS/G/AX8 (31.6% DS and 8%AX).

Additionally, in order to analyze the influence of the presence of AX, a control set of starch and starch–gluten (without AX) systems was prepared: (1) NS/4 (4.1% DS), (2) NS/8 (4.1% DS), (3) DS/4 (31.6% DS), (4) DS/8 (31.6% DS), and (1) NS/G/4 (4.1% DS), (2) NS/G/8 (4.1% DS), (3) DS/G/4 (31.6% DS), (4) DS/G/8 (31.6% DS) (Table 1).

For testing, the solid samples were dispersed in water (w) or sucrose 50% *w*/*w* solution (s) to study the effect of AX and DS on batter-type products in which there is a high amount of sucrose.

### 2.5. Pasting Profile

The pasting properties of samples were determined using a Rapid Visco Analyzer (RVA series 4500, Perten Instruments, Stockholm, Sweden). Samples (3.5 g) and water or sucrose 50% *w*/*w* solution (25 g) were weighed in an RVA aluminum canister. The starch concentration was 11.9% *w*/*w* (starch/AX4), 11.4% *w*/*w* (starch/AX8), 10.8% *w*/*w* (starch/gluten/AX4), and 10.4% *w*/*w* (starch/gluten/AX8). In the case of the control samples, the starch concentration was the same as for the samples with AX.

Sample pasting profiles were analyzed using the Standard 1 method profile (Thermocline for Windows TCW 3.17.3.509 software, RVA series 4500, Perten instruments). Peak viscosity (PV), breakdown (BD), final viscosity (FV), and setback (SB) were determined from the pasting profiles. Additionally, other parameters were defined to describe the pasting profiles of the samples dispersed in the sucrose 50% (*w*/*w*) solution: viscosity at the end of the constant-temperature period (temperature profile step 3) (FPCV) and maximum viscosity reached in the test (MV) [11]. All measurements were performed in duplicate.

### 2.6. Differential Scanning Calorimetry (DSC)

Samples (~10 mg) and water (~30 µL) or sucrose 50% (*w*/*w*) solution (~30 µL) were weighed in an aluminum pan (100 µL). The starch concentration was 24.2% *w*/*w* (starch/AX4), 23.5% *w*/*w* (starch/AX8), 22.4% *w*/*w* (starch/gluten/AX4), and 21.6% *w*/*w* (starch/gluten/AX8). In the case of the control samples, the starch concentration was the same as for the samples with AX. The aluminum pans were sealed and equilibrated at room temperature for at least 12 h before heating analysis to equilibrate the solids-water mixture. Thermal studies were performed with a DSC823e Calorimeter and thermograms were evaluated by STARe Default DB V9.00 software (Mettler Toledo, Greifensee, Switzerland). The DSC analyzer was calibrated using indium and an empty aluminum pan was used as a reference. The samples were held at 25 °C for 5 min and heated from 25 to 110 °C at 5 °C/min. Onset temperature (T_O_ (°C)), peak width at half height (PW (°C)), and change in enthalpy referred to gelatinization (ΔH_g_ (J/g starch)) were determined. All measurements were carried out in triplicate.

### 2.7. Statistical Analysis

Data were statistically treated using analysis of variance (ANOVA). The means were compared using an LSD Fischer test at a significance level of 0.05. The relationship between the variables was studied by principal component analysis (PCA). INFOSTAT-version 2011 statistical software (Facultad de Ciencias Agropecuarias, UNC, Córdoba, Argentina) was used for statistical analysis.

## 3. Results and Discussion

### 3.1. Pasting Properties

The samples dispersed in water had different pasting profiles compared to those dispersed in sucrose solution (Figures S1 and S2). It was observed that the profiles of the samples in sucrose solution showed higher viscosities than the samples dispersed in water throughout the entire temperature protocol of the test (heating, holding, and cooling). The curves of the samples in the water showed that, after reaching the PV, there was a viscosity decrease. On the other hand, during heating and holding time at maximum temperature, the pasting profiles in sucrose solution showed a continuous increase in viscosity. The maximum starch paste viscosity was delayed due to the delayed gelatinization of starch [11]. This may be due to the lesser fragmentation of the starch granules and/or fewer starch molecules leaching from the granules [12].

- Water as a dispersion medium

The addition of 4 and 8% AX to the native starch did not change (Table 2) the PV and FV values compared to their controls. However, the SB values increased by 34% and 58% with the addition of 4% and 8% AX, respectively. Conversely, when the DS content was increased from 4.1 to 31.6%, the addition of 4% AX did not produce significant differences, but the addition of 8% AX caused a 43.1% increase in PV and 71.6% in the FV. Moreover, the SB values increased by 81% and 142% with the addition of 4 and 8% AX, respectively. Therefore, PV, FV, and SB were found to be dependent on an AX concentration when the DS increased.

In the presence of gluten, the addition of AX did not alter the PV and FV values of the native starch. However, the addition of 4% and 8% AX in the system with 31.6%DS caused increases of 14.8% and 27.4% in the PV, 48% and 72% in the FV, and 68% and 123% in the SB compared to their controls (Table 2), respectively. These results indicate that, in systems with a higher DS content, gluten might affect the pasting parameters at lower AX levels. Conversely, gluten appeared to leave the AX effect unchanged at higher AX levels.

Based on the behavior of the samples in water, it can be inferred that AX levels only affect the viscosity properties of systems with high DS contents, both with and without gluten.

- Sucrose solution as a dispersion medium

The additions of 4% and 8% AX to the native starch produced decreases of 18.7% and 24% in the FPCV and increases of 6.5% and 53.4% in the FV, respectively (Table 3). Arabinoxylans significantly modified the pasting profiles of samples in sucrose, while these macromolecules did not cause any change in the same samples in water. In contrast, the addition of 4% AX did not significantly change FPCV value, as opposed to 8% AX, which produced a slight increase in FPCV. The addition of 4% and 8% of AX produced an increase of 43.1% and 185% in FV, respectively. The system’s viscosity was dependent on the AX concentration. Compared to native starch systems, the addition of AX resulted in substantially larger increases in pasting parameters for systems with higher DS levels. Furthermore, the rise in FV induced by 8% AX was significantly greater in sucrose-dispersed systems compared to those in water.

In the presence of gluten, the addition of AX affected the pasting profile of the system with native starch, unlike the behavior observed in the same samples dispersed in water. The incorporation of 4% and 8% of AX increased to 22.3% and 101% FV, respectively. In the samples with a higher DS content, the addition of 4% and 8% AX produced an increase of 25.7% and 112.2% in FPCV, and 78.9% and 268% in the FV, respectively. Additionally, the increments in FV caused by the addition of AX were higher in sucrose-dispersed systems compared to those in water. The presence of gluten intensified the effect of AX.

In the sucrose solution, the effect of the AX depended on its concentration, DS content, and the presence of gluten.

Increasing the content of DS led to a higher viscosity in samples dispersed in water or in sucrose solution when the AX was added. The dependence on the DS content in the systems resulting from the effect of the AX level could be related to the interaction between AX and swollen starch granules. Starch granules with higher damaged levels are, thus, suggested to set more interactions with AXs by hydrogen bonds than starch granules with lower damaged levels. It is known that the mechanical damage of starch granules increases the exposition of hydroxyl groups [13], which in turn increases interactions via hydrogen bonding with the hydroxyl groups of the AXs. Likewise, Hussain et al. [14] suggested that the starch–polysaccharide (gum)–water system is biphasic with the presence of polysaccharides in a continuous liquid phase. Before heating, soluble polysaccharides were present in a continuous phase at a certain concentration, but during the heating process, this concentration increased due to reductions in water caused by the absorption of liquid by starch granules. Conversely, amylose molecules leached from the starch granules during gelatinization occupied the volume and increased the concentration of water-soluble molecules in the liquid phase. The viscosity increased due to the reduction in free water molecules caused by starch granules swelling and the increase in the concentration of water-soluble molecules. Consequently, interactions among polysaccharides, solubilized starch, and swollen starch granules produced a higher viscosity on the paste. Therefore, as the concentration of AX in the sample increased, the viscosity also increased.

During the cooling process, the SB value indicated the extent to which starch paste retrograded, particularly the recrystallization and rearrangement of amylose molecules. [15]. Furthermore, the gel-forming ability of wheat arabinoxylans could be attributed to their structural characteristics, including their molecular weight, branching degree, and the presence of certain functional groups. In the presence of water, AX can interact with it and with itself to form a gel-like network, leading to the formation of a three-dimensional structure. This gelation is often influenced by factors such as the concentration, temperature, and pH. Higher FV and SB values could be related to the AX molecules’ association as a result of a higher AX proportion in the system. Likewise, AX molecules may interact with amylose chains, contributing to system interconnection. In addition, in these water-dispersed systems, gluten caused smaller SB increases for starch mixtures with a higher damage content. In this case, the gluten could be acting as a barrier that prevented reassociations between the amylose chains in the gelation process.

The results indicated that the effect of AX on the pasting profiles depended on the dispersing medium. The impact of the AX level on systems’ viscosity was more meaningful when a 50% sucrose solution was used as a dispersion medium. Considering that sucrose molecules were found in a continuous liquid phase, water was even more unavailable, which directly affected the starch viscous properties. As for the samples evaluated in water, the increase in DS content produced greater increases in viscosities dispersions. However, in the sucrose solution, the addition of AX increased the viscosity of the native starch systems. Yan et al. [15] studied the effect of wheat bran arabinoxylan on wheat starch gelatinization using 0.5% (*w*/*w*) arabinoxylan dispersions. They found increments of 84% and 71% in PV and FV due to the incorporation of AX in concentrations similar to those used in this work. The authors attribute these effects to the stronger interactions between AXs with lower branching degrees and swollen starch granules [16] or leached starch molecules [17]. The PV is known to be influenced by the amount of amylose leaching, amylose–lipid complex formation, friction between swollen granules, granule swelling, and competition for free water between leached amylose and remaining ungelatinized granules [18]. Amylose leaching from starch granules is an important parameter in starch gelatinization, and it is related to amylose dilution and swelling suppression [19]. Xie et al. [20] studied the effect of arabinoxylans with different molecular weights on wheat starch gelling properties, and found that AXs decreased the concentration of leached amylose. Moreover, the concentration of leached amylose decreased as the concentration of AX increased, indicating that the addition of AX significantly reduced the starch leaching. Other studies have shown that polysaccharides can bind more water and increase the viscosity of starch in the continuous phase, thereby preventing the leaching of amylose molecules from the starch granules [21,22]. Therefore, an increase in FPCV (an equivalent of PV in sucrose solution) could be related to a lower amount of leached amylose, due to the presence of AX in the medium and to stronger and more resilient swollen starch granules.

The anti-plasticizing effect of sugars has been explained by a free volume decrease, relative to the plasticizing action of water alone. The reduced molecular mobility of the amorphous chain domains would then result in a higher gelatinization temperature [23]. Moreover, the presence of sugar molecules affects the molecular mobility of water in high-sucrose solutions. A previous study [24] showed that, at high sucrose concentrations, the sugar molecules distort the water hydrogen bond network and increase the rotational mobility of the bulk water compared to pure water. Additionally, the hydration properties and proton exchange in aqueous sugar solutions indicate a decrease in water mobility at high sugar concentrations.

Finally, the presence of gluten caused a marked increase in viscosity parameters when sucrose was present. Si et al. [25] conducted research on the interactions between gluten and the water-unextractable AX during thermal treatment. This study found that AX could reduce gluten solubility in urea solution at 25 °C, but improve it at 60 °C and 95 °C. This was due to the hydrophobic interactions and dynamic rearrangement during the heating process, which helped stabilize the gluten molecular chains [26]. Additionally, intrinsic fluorescence spectra analysis showed that AX weakened the hydrophobic interactions at 25 °C, while enhancing them at 95 °C due to the rearrangement of molecular interactions during heating. This indicates that AX can affect the denaturation and/or aggregation of gluten by expanding the hydrophobic core of proteins during heating through its hydrophobic side chains [27]. This rearrangement of hydrophobic interactions between AX and gluten could cause an increase in viscosity.

### 3.2. Differential Scanning Calorimetry Studies

The gelatinization parameters, including ΔH_g_, T_O_, and PW of samples calculated from the DSC thermograms are summarized in Table 4 and Table 5. As a general behavior, when comparing the samples dispersed in water vs. sucrose solution, sucrose was observed to cause a significant shift in the gelatinization peak to higher temperatures (from ~50 to ~80 °C). This can be the result of the competition between starch and sugar for water (reduced water availability, depression of water activity), sugar–starch interactions (sugars may act as cross-linking agents stabilizing the polymer chains in the granular structure via intermolecular H-bonding), and an anti-plasticization mechanism by the sugar–water cosolvent [28]. Moreover, the increase in the DS content caused decreases in ΔH_g_ and T_O_ values during heating due to the spontaneous gelatinization of starch in cold water, as has been reported in previous studies [29,30,31].

- Water as a dispersion medium

The ΔH_g_ value decreased by 25.4% and 29.7% as a consequence of adding 4% and 8% AX to native starch systems compared to their controls (Table 3), respectively. In addition, the T_O_ value was modified by adding AX: in the case of native starch, adding 4% and 8% AX caused a significant delay in the gelatinization process of 1.3 °C for 4% AX and 1.9 °C for 8%. In contrast, when increasing the DS content from 4.1% to 31.6%, adding 4% AX did not cause significant differences in the ΔH_g_ value. Only when 8% AX was added, was a significant increase of 18% observed in the ΔH_g_ value. However, the effect was the opposite when increasing the DS content. That is, the gelatinization peak shifted to lower temperatures: only the addition of 8% of AX caused a significant decrease of 1.2 °C.

When gluten was present, the impact of AX was comparable to that of starch (native and damaged)/AX systems. In the NS/G system, the inclusion of 4% and 8% AX resulted in a reduction of 17.5% and 21.9% in the ΔH_g_ value. Conversely, T_O_ was affected by the addition of AX. When adding 4% and 8% AX to the NS/G systems, a significant increase of 0.8 and 1.6 °C in T_O_ values was observed. Moreover, the presence of gluten did not modify the effects caused by AXs on the thermal properties of starch when water was used as a solvent. The addition of 4% AX to DS/G systems did not significantly alter the ΔH_g_ value. However, when 8% AX was added, there was a 27.9% increase in the value of this parameter. When the DS content increased from 4.1% to 31.6%, the addition of AX did not cause significant changes in the T_O_ value for the DS/G systems.

- Sucrose solution as a dispersion medium

The addition of 4% and 8% AX to native starch produced a significant decrease of 11% and 49% in the ΔH_g_ values (Table 4). Furthermore, there was a significant increase in the T0 values: when adding 4% and 8% of AX, the temperatures increased by 0.5 and 2.7 °C, respectively. As the DS content increased, ΔH_g_ values decreased by 29.4% and 15.6% with the addition of 4% and 8% AX, respectively. In the samples with the highest DS content, the addition of AX did not significantly modify the T_O_.

In the presence of gluten, the addition of 4 and 8% AX caused a significant decrease of 27.3% and 14% in the ΔH_g_ values, respectively. In contrast, when adding 4% and 8% of AX, T_O_ decreased 1.5 °C and 2.4 °C. The addition of 4% and 8% AX to DS/G systems in sucrose did not significantly alter the ΔH_g_ values. Moreover, a T_O_ increase of 1.8 °C and 2.9 °C was observed when adding 4% and 8% of AX, respectively.

As regards the analysis of systems with native starch, we noticed a decrease in the ΔH_g_ values and an increase in T_O_, in the presence of gluten and water or sucrose solution as the dispersing solvent.

Other authors also observed a decrease in ΔH_g_ values due to the addition of AX. They attributed this effect to the restriction and reduction in water availability caused by the AX (1% and 2% of AX) [32]. Additionally, the strong hydrogen bonding between AX and leached amylose restrict the internal association between amylose and amylopectin (1.5% of AX) [15]. Several researchers examined how polysaccharides (hydrocolloids) affected starch gelatinization, and they observed that the addition of such molecules resulted in a ΔH_g_ decrease. Kim et al. [33] provided an explanation of this trend by suggesting that the decrease in ΔH_g_ caused by the addition of 0.3% and 0.6% galactomannans could be due to the polysaccharides’ ability to reduce water availability. This reduction in water availability leads to the partial gelatinization of the crystalline regions in the starch granules [34].

Based on the available evidence, it appears that the effect of AX cannot be solely attributed to a water restriction. Kruger, Ferrero, and Zaritzky [35] suggested that a small reduction in ΔH_g_ when xanthan, or guar gum, or sodium alginate (at 1%), and excess water are present is due to the slower heat transfer rates and reduced mobility of water molecules.

According to Ratnayake and Jackson [36], the starch transition phase is a gradual process and cannot be considered sudden or quick. The process takes place in three stages, during which the following structural events occur: (1) starch granules absorb water, leading to an increase in starch polymer mobility in the amorphous regions, especially amylose molecules; (2) starch polymers in the amorphous regions rearrange, often forming new intermolecular interactions; and (3) with increasing hydrothermal effects, the polymers become more mobile, lose their intermolecular interactions, and overall granular structure. The energy absorbed by granules melts crystallite structures during gelatinization as well as facilitates the formation of new bonds among molecules at lower temperatures, a process called ‘rearrangement’. During this structural reordering process, an array of new molecular rearrangements and bonds with varying stabilities is formed. This suggests that the ΔH_g_ decrease in the presence of AXs may be related to the formation of new and more stable bonds during the second gelatinization phase. These polymers may interact with starch molecules through hydrogen bonding or covalent bonds. A similar theory was proposed by Biliadieris et al. [37]: the starch gelatinization process is composed of three phases: partial melting, recrystallization, and final melting. During gelatinization, the authors proposed that a three-phase theory was a better fit for nonequilibrium crystallite melting. They also observed that the DSC transitions did not accurately reflect the initial phase transitions of a starch semicrystalline structure, but rather represented a composite of melting and reorganization that occurred during thermal analysis. Its close association with some parts of crystalline domains restricted the polymer mobility in the bulk amorphous region. Furthermore, they suggested that the intracrystalline amorphous region did not exhibit “normal” amorphous characteristics due to the strains caused by crystalline domains. The presence of AX caused a ΔH_g_ decrease, suggesting that the recrystallization phase would be more significant with these biopolymers. Arabinoxylans may interact with the starch molecules through different types of bonds, stabilizing them and causing recrystallization during gelatinization. As it is known that recrystallization is an exothermic process, in this case, this energetic component would be more important when AXs are added.

Arabinoxylans caused a delay in the disorganization of the starch amorphous regions during gelatinization, as reflected in T_O_ values. The delay of gelatinization on the addition of polysaccharides can be interpreted as a result of the limited water availability for starch gelatinization owing to their greater hydration capacity [38]. The polysaccharide structure with a higher degree of branching could provide spatially confined environments for water sorption, leading to a higher hydration capacity with a much more well-ordered network water structure [39]. Arabinoxylans could readily hydrate and consequently reduce water availability during the wheat starch gelatinization. Therefore, AXs with higher Mw and branching degrees had a more pronounced inhibition effect on starch gelatinization as compared to those with a lower Mw and branching degree [15]. According to Shahzad et al. [40], the addition of arabic, xanthan, cress seed, fenugreek, flaxseed, and okra to starch can cause a shift in T_O_ to higher temperatures. This shift may be due to the competition between gums and starch granules for water, which delays water migration into the starch [41].

The systems containing starch with a higher level of damage behaved very differently from those previously described: the addition of AX to starch (in the absence and presence of gluten) produced an increase in ΔH_g_ (8% of AX) and a decrease in T_O_ when water was used as solvent. However, in sucrose solution, the ΔH_g_ showed small decreases while the T_O_ showed no clear trends.

Other authors also found that the addition of polysaccharides increased ΔH_g_. Lee et al. [42] proposed that the ΔH_g_ increases due to the addition of 0.3% xanthan to sweet potato starch can be explained by a stronger association between the starch and xanthan. This association could restrict the movement of the starch chains, requiring more heat energy for gelatinization and causing the ΔH_g_ to increase. Considering that, before heating, DS spontaneously gelatinizes in cold water, AX can interact with the exposed hydroxyl groups on the gelatinized starch granules and the leached amylose chains, thus increasing ΔH_g_. On the other hand, Shazhad et al. [40] attributed the increase in ΔH_g_ observed when adding flaxseed at 0.5% gum concentration and flaxseed, fenugreek, and cress seed at 2% gum concentration to a delay the migration of water towards the starch granules during gelatinization. This delay led to a higher energy requirement for the process.

### 3.3. Principal Components Analysis (PCA) and Cluster Analysis

**Figure 1:**
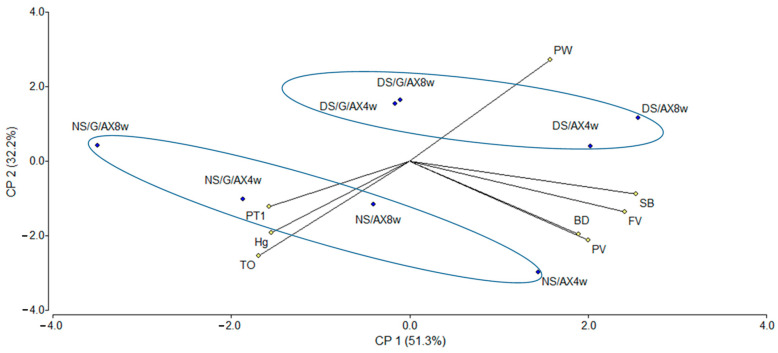
Principal component analysis of the RVA (PV, BD, SB, FV, and Pt) and DSC (ΔH_g_, T_O_, and PW) variables. Samples and controls in water.

**Figure 2:**
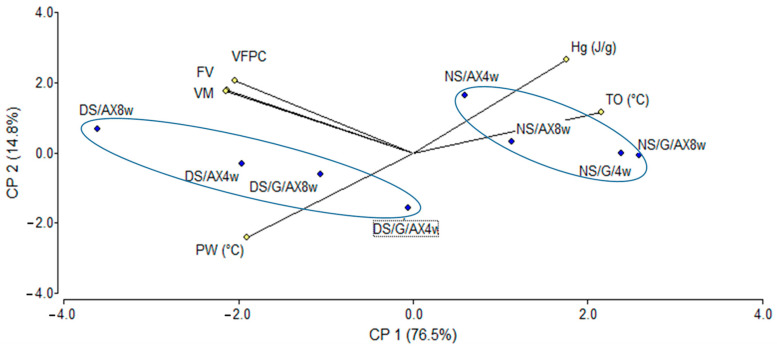
Principal component analysis of the RVA (FV, MV, and FPCV) and DSC (ΔH_g_, T_O,_ and PW) variables. Samples and controls in sucrose 50% *w*/*w* solution.

PCAs explaining correlations among the pasting and thermal properties of starch–AX systems in water and in sucrose solution are shown in Figure 1 and Figure 2. PCA was performed in water and in sucrose solution separately to evaluate the impact of parameters on sample variability.

The bi-plot of PCA indicated that 83.5% of the total variability of water samples was represented. The projection of PC1 and PC2 explained 51.3% and 32.2%, respectively, of the sample’s variability. PC1 was characterized by PW, PV, DD, SB, and FV on the positive side, and ΔH_g_, T_O_, and PT on the negative one. PW was negatively correlated to ΔH_g_, T_O_, and PT. PC1 separated samples containing high levels of DS and gluten (negative side of PC1) and without gluten (positive side of PC1). However, no distinction regarding the gluten presence of samples containing low levels of DS was found. The samples in water were separated by PC2 based on their DS content. Samples with 4.1% DS were in the negative axis region, while samples with 31.6% DS were placed in the positive axis region.

The bi-plot obtained through PCA showed that the samples in sucrose solution had a total variability of 91.3%. The projection of PC1 and PC2 explained 76.5% and 14.8%, respectively, of the sample’s variability. PC1 was characterized by ΔH_g_, and T_O_ on the positive side, and PW, VM, FV, and VFPC on the negative one. PW was negatively correlated to ΔH_g_ and T_O_. The samples in sucrose solution were separated by PC1 according to the DS content: samples with 4.1%DS were in the positive region of the axis while samples with 31.6%DS were placed in the positive region of the axis. No clear distinction regarding gluten presence was found.

These results confirm that DS had greater effects than AX on the pasting and thermal properties of starch. Also, it is suggested that gluten had a higher preponderance in systems dispersed in sucrose solution than in systems dispersed in water.

## 4. Conclusions

This study analyzed the impact of DS content and AXs on the wheat starch and wheat starch–gluten system thermal properties using water and a concentrated sucrose solution as solvents.

The addition of AX affected the pasting properties, particularly when the DS content was high, regardless of the presence or absence of gluten. In the case of water-dispersed samples, the effects of AX were not significant for systems containing native starch. The samples with a higher DS content showed increased viscosity profiles because of the addition of AXs. When both gluten and sucrose were present together, the viscosity increased even more. In addition, the changes in viscosity caused by the addition of AX were found to be dependent on the AX concentration. The results indicated that this increase in viscosity might be attributed to the hydrogen bond interactions between arabinoxylan molecules and the exposed hydroxyl groups of the DS. Moreover, sucrose generates an antiplasticizing effect on starch chains and also competes for water with AXs and starch. This leads to a further increase in viscosity due to an increase in the volume fraction of starch in the dispersion.

The DSC technique helped us examine how starch gelatinized when AX, gluten, and sucrose were present, while also changing the amount of DS. This research showed that adding AX to starch and starch–gluten in water caused a decrease in the change in the enthalpy and onset temperature of starch gelatinization. However, when the DS content increased, the trend was reversed. In contrast, when sucrose was present, AX led to an increase in the change in the enthalpy and onset temperature of starch gelatinization for both starch and starch–gluten systems.

In general, our results demonstrated that DS had greater effects than AX on the pasting and thermal properties of starch. Similarly, gluten had a higher influence on the systems dispersed in sucrose solution than in systems dispersed in water.

These results are relevant in the study of batter products, which contain high concentrations of sucrose. Arabinoxylans in conjunction with damaged starch affect the batter viscous properties, which could alter the quality of products and their processing, including phenomena such as the formation of bubbles in the batter and the spongy texture of the final product.

## Figures and Tables

**Table 1:** Samples preparation.

System	Medium			
	Water		Sucrose	
	Samples			
	Control	AX	Control	AX
Starch	NS/4w	NS/AX4w	NS/4s	NS/AX4s
	NS/8w	NS/AX8w	NS/8s	NS/AX8s
	DS/4w	DS/AX4w	DS/4s	DS/AX4s
	DS/8w	DS/AX8w	DS/8s	DS/AX8s
Starch–Gluten	NS/G/4w	NS/G/AX4w	NS/G/4s	NS/G/AX4s
	NS/G/8w	NS/G/AX8w	NS/G/8s	NS/G/AX8s
	DS/G/4w	DS/G/AX4w	DS/G/4s	DS/G/AX4s
	DS/G/8w	DS/G/AX8w	DS/G/8s	DS/G/AX8s

Abbreviations: NS: native starch, DS: damaged starch, AX: arabinoxylans, G: gluten, w: water, s: sucrose solution.

**Table 2:** Effect of AX and DS on the starch and starch–gluten mixture pasting parameters in water.

Sample	PV (cP)	BD (cP)	FV (cP)	SB (cP)	Pt (°C)
NS/4w	3887 ± 148 ^a^	705 ± 7 ^a^	4771 ± 147 ^a^	1589 ± 6 ^a^	85.9 ± 0.5 ^a^
NS/AX4w	3646 ± 54 ^a^	1017 ± 18 ^b^	4754 ± 23 ^a^	2125 ± 58 ^b^	87.6 ± 0.6 ^a^
NS/8w	2938 ± 141 ^a^	587 ± 63 ^a^	3477 ± 182 ^a^	1127 ± 104 ^a^	88.8 ± 0.0 ^b^
NS/AX8w	2945 ± 78 ^a^	795 ± 102 ^a^	3935 ± 57 ^a^	1785 ± 123 ^b^	82.7.3 ± 1.8 ^a^
NS/G/4w	3082 ± 68 ^a^	630 ± 8 ^a^	3731 ± 67 ^a^	1278 ± 7 ^a^	88.8 ± 0.0 ^a^
NS/G/AX4w	2994 ± 55 ^a^	585 ± 61 ^a^	3757 ± 91 ^a^	1348 ± 97 ^a^	89.2 ± 0.5 ^a^
NS/G/8w	2360 ± 20 ^a^	460 ± 2 ^a^	2882 ± 4 ^a^	981 ± 14 ^a^	90.5 ± 0.0 ^a^
NS/G/AX8w	2342 ± 153 ^a^	374 ± 151 ^a^	3133 ± 168 ^a^	1164 ± 165 ^a^	90.0 ± 0.6 ^a^
DS/4w	2995 ± 31 ^a^	453 ± 77 ^a^	3726 ± 20 ^a^	1184 ± 26 ^a^	84.8 ± 0.0 ^a^
DS/AX4w	3077 ± 164 ^a^	906 ± 11 ^b^	4315 ± 224 ^a^	2143 ± 71 ^b^	86.8 ± 0.6 ^b^
DS/8w	2266 ± 33 ^a^	518 ± 62 ^a^	2727 ± 94 ^a^	851 ± 0 ^a^	88.0 ± 1.0 ^b^
DS/AX8w	3243 ± 6 ^b^	621 ± 34 ^b^	4679 ± 156 ^b^	2057 ± 117 ^b^	67.7 ± 2.3 ^a^
DS/G/4w	2426 ± 26 ^a^	464 ± 21 ^a^	2839 ± 30 ^a^	878 ± 18 ^a^	88.4 ± 0.6 ^a^
DS/G/AX4w	2785 ± 6 ^b^	643 ± 23 ^b^	3618 ± 21 ^b^	1476 ± 37 ^b^	88.0 ± 0.0 ^a^
DS/G/8w	1776 ± 1 ^a^	380 ± 170 ^a^	2134 ± 8 ^a^	738 ± 1 ^a^	90.5 ± 0.1 ^a^
DS/G/AX8w	2629 ± 83 ^b^	611 ± 182 ^a^	3666 ± 148 ^b^	1648 ± 247 ^b^	79.8 ± 13.8 ^a^

Abbreviations: PV: peak viscosity, BD: breakdown, FV: final viscosity, SB: setback, Pt: pasting temperature. Statistical comparisons were made as a function of arabinoxylans presence. Values followed by different letters in the same column are significantly different (*p* < 0.05).

**Table 3:** Effects of AX and DS on the starch and starch–gluten mixture pasting parameters in sucrose 50% *w*/*w* solution.

Sample	FV (cP)	MV (cP)	FPCV (cP)
NS/4s	11,539 ± 164 ^a^	11,692 ± 190 ^a^	9959 ± 86 ^b^
NS/AX4s	12,293 ± 90 ^b^	12,315 ± 113 ^a^	8095 ± 124 ^a^
NS/8s	7477 ± 91 ^a^	7708 ± 104 ^a^	7210 ± 6 ^b^
NS/AX8s	11,470 ± 481 ^b^	11,470 ± 481 ^b^	5478 ± 139 ^a^
NS/G/4s	6177 ± 120 ^a^	6371 ± 120 ^a^	5809 ± 100 ^b^
NS/G/AX4s	7556 ± 194 ^b^	7561 ± 196 ^b^	5090 ± 190 ^a^
NS/G/8s	4085 ± 192 ^a^	4230 ± 173 ^a^	3771 ± 179 ^a^
NS/G/AX8s	8210 ± 109 ^b^	8210 ± 109 ^b^	4040 ± 114 ^a^
DS/4s	9549 ± 0 ^a^	9716 ± 27 ^a^	8560 ± 29 ^a^
DS/AX4s	13,661 ± 176 ^b^	13,772 ± 319 ^b^	8806 ± 281 ^a^
DS/8s	6594 ± 51 ^a^	6660 ± 45 ^a^	5868 ± 88 ^a^
DS/AX8s	18,781 ± 1049 ^b^	18,794 ± 1049 ^b^	9768 ± 346 ^b^
DS/G/4s	5248 ± 238 ^a^	5301 ± 250 ^a^	4578 ± 281 ^a^
DS/G/AX4s	9387 ± 246 ^b^	9390 ± 246 ^b^	5755 ± 37 ^b^
DS/G/8s	3611 ± 9 ^a^	3639 ± 26 ^a^	3070 ± 25 ^a^
DS/G/AX8s	13,276 ± 147 ^b^	13,377 ± 289 ^b^	6515 ± 100 ^b^

Abbreviations: FV: final viscosity, MV: maximum viscosity, FPCV: viscosity at the end of constant temperature period. Statistical comparisons were made as a function of the presence of arabinoxylans. Values followed by different letters in the same column are significantly different (*p* < 0.05).

**Table 4:** Effect of AX and DS on the starch and starch–gluten mixtures’ gelatinization parameters in water measured by DSC.

Sample	ΔH_g_ (J/g)	T_O_ (°C)	PW (°C)
NS/4w	8.34 ± 0.23 ^b^	55.2 ± 0.1 ^a^	7.1 ± 0.0 ^a^
NS/AX4w	6.22 ± 0.02 ^a^	56.4 ± 0.3 ^b^	6.6 ± 0.5 ^a^
NS/8w	8.52 ± 0.14 ^b^	55.2 ± 0.0 ^a^	6.9 ± 0.1 ^a^
NS/AX8w	5.99 ± 0.55 ^a^	57.1 ± 0.2 ^b^	6.8 ± 0.3 ^a^
NS/G/4w	8.08 ± 0.34 ^b^	55.5 ± 0.0 ^a^	7.0 ± 0.1 ^a^
NS/G/AX4w	6.67 ± 0.36 ^a^	56.3 ± 0.4 ^a^	6.7 ± 0.5 ^a^
NS/G/8w	8.27 ± 0.08 ^b^	55.4 ± 0.1 ^a^	7.3 ± 0.0 ^b^
NS/G/AX8w	6.46 ± 0.35 ^a^	57.0 ± 0.0 ^b^	7.1 ± 0.1 ^a^
DS/4w	4.81 ± 0.08 ^a^	51.0 ± 0.4 ^a^	9.5 ± 0.2 ^a^
DS/AX4w	5.10 ± 0.44 ^a^	50.1 ± 0.5 ^a^	10.5 ± 0.2 ^b^
DS/8w	4.60 ± 0.09 ^a^	51.1 ± 0.0 ^b^	8.9 ± 0.8 ^a^
DS/AX8w	5.43 ± 0.04 ^b^	50.0 ± 0.3 ^a^	11.0 ± 0.4 ^a^
DS/G/4w	4.10 ± 0.38 ^a^	51.5 ± 0.2 ^a^	9.3 ± 0.3 ^a^
DS/G/AX4w	4.64 ± 0.02 ^a^	51.0 ± 0.4 ^a^	9.8 ± 0.3 ^a^
DS/G/8w	4.68 ± 0.13 ^a^	51.3 ± 0.1 ^b^	9.5 ± 0.2 ^a^
DS/G/AX8w	5.99 ± 0.28 ^b^	50.6 ± 0.2 ^a^	10.4 ± 0.2 ^b^

Abbreviations: ΔH_g_: change in enthalpy referred to gelatinization, T_O_: onset temperature, PW: peak width. Statistical comparisons were made as a function of the presence of arabinoxylans. Values followed by different letters in the same column are significantly different (*p* < 0.05).

**Table 5:** Effect of AX and DS on the starch and starch–gluten mixtures’ gelatinization parameters in sucrose 50% *w*/*w* solution measured by DSC.

Sample	ΔH_g_ (J/g)	T_O_ (°C)	PW (°C)
NS/4w	8.34 ± 0.23 ^b^	55.2 ± 0.1 ^a^	7.1 ± 0.0 ^a^
NS/AX4w	6.22 ± 0.02 ^a^	56.4 ± 0.3 ^b^	6.6 ± 0.5 ^a^
NS/8w	8.52 ± 0.14 ^b^	55.2 ± 0.0 ^a^	6.9 ± 0.1 ^a^
NS/AX8w	5.99 ± 0.55 ^a^	57.1 ± 0.2 ^b^	6.8 ± 0.3 ^a^
NS/G/4w	8.08 ± 0.34 ^b^	55.5 ± 0.0 ^a^	7.0 ± 0.1 ^a^
NS/G/AX4w	6.67 ± 0.36 ^a^	56.3 ± 0.4 ^a^	6.7 ± 0.5 ^a^
NS/G/8w	8.27 ± 0.08 ^b^	55.4 ± 0.1 ^a^	7.3 ± 0.0 ^b^
NS/G/AX8w	6.46 ± 0.35 ^a^	57.0 ± 0.0 ^b^	7.1 ± 0.1 ^a^
DS/4w	4.81 ± 0.08 ^a^	51.0 ± 0.4 ^a^	9.5 ± 0.2 ^a^
DS/AX4w	5.10 ± 0.44 ^a^	50.1 ± 0.5 ^a^	10.5 ± 0.2 ^b^
DS/8w	4.60 ± 0.09 ^a^	51.1 ± 0.0 ^b^	8.9 ± 0.8 ^a^
DS/AX8w	5.43 ± 0.04 ^b^	50.0 ± 0.3 ^a^	11.0 ± 0.4 ^a^
DS/G/4w	4.10 ± 0.38 ^a^	51.5 ± 0.2 ^a^	9.3 ± 0.3 ^a^
DS/G/AX4w	4.64 ± 0.02 ^a^	51.0 ± 0.4 ^a^	9.8 ± 0.3 ^a^
DS/G/8w	4.68 ± 0.13 ^a^	51.3 ± 0.1 ^b^	9.5 ± 0.2 ^a^
DS/G/AX8w	5.99 ± 0.28 ^b^	50.6 ± 0.2 ^a^	10.4 ± 0.2 ^b^

Abbreviations: ΔH_g_: change in enthalpy referred to gelatinization, T_O_: onset temperature, PW: peak width. Statistical comparisons were made as a function of the presence of arabinoxylans. Values followed by different letters in the same column are significantly different (*p* < 0.05).

## Data Availability

The original contributions presented in the study are included in the article/supplementary material, further inquiries can be directed to the corresponding author.

## Supplementary Materials

The following supporting information can be downloaded at: https://www.mdpi.com/article/10.3390/foods13050689/s1, Figure S1: Effect of arabinoxylans and damaged starch on the starch and starch-gluten pasting profiles in water; Figure S2: Effect of arabinoxylans and damaged starch on the starch and starch-gluten pasting profiles in water.

## References

[1] Ogunsona E., Ojogbo E., Mekonnen T. (2018). Advanced material applications of starch and its derivatives. Eur. Polym. J..

[2] Izydorczyk M.S., Biliaderis C. (1995). Cereal Arabinoxylans: Advances in structure and physicochemical properties. Carbohydr. Polym..

[3] Zhang C., Wang P., Yang J., Lu Z., Zhao H., Lu F. (2019). Oxidative crosslinking of water-extractable wheat arabinoxylans by recombinant lipoxygenase and its effect on bread properties. Food Sci. Technol..

[4] Hernández-Pinto F.J., Miranda-Medina J.D., Natera-Maldonado A., Vara-Aldama O., Ortueta-Cabranes M.P., Vázquez del Mercado-Pardiño J.A., El-Aidie S.A.M., Siddiqui S.A., Castro-Muñoz R. (2024). Arabinoxylans: A review on protocols for their recovery, functionalities and roles in food formulations. Int. J. Biol. Macromol..

[5] Moza J., Gujral H.S. (2017). Influence of barley non-starchy polysaccharides on selected quality attributes of sponge cakes. Food Sci. Technol..

[6] Teobaldi A.G., Barrera G.N., Severini H., Ribotta P.D. (2023). Influence of damaged starch on thermal and rheological properties of wheat starch and wheat starch-gluten systems in water and sucrose. J. Sci. Food Agric..

[7] Zhou S., Liu X., Guo Y., Wang Q., Peng D., Cao L. (2010). Comparison of the immunological activities of arabinoxylans from wheat bran with alkali and xylanase-aided extraction. Carbohydr. Polym..

[8] Anderson C., Simsek S. (2019). Mechanical profiles and pographical properties of films made from alkaline extracted arabinoxylans from wheat bran, maize, bran, or dried distillers grain. Food Hydrocoll..

[9] Hashimoto S., Shogren M.D., Pomeranz Y. (1987). Cereal pentosans: Their estimation and significance. I. Pentosans in wheat and milled wheat products. Cereal Chem..

[10] American Association of Cereal Chemists International (AACC) (2000). Approved Methods of Analysis.

[11] Teobaldi A.G., Barrera G.N., Sciarini L.S., Ribotta P.D. (2021). Pasting, gelatinization, and rheological properties of wheat starch in the presence of sucrose and gluten. Eur. Food Res. Technol..

[12] Kim C.S., Walker E. (1992). Changes in starch pasting properties due to sugars and emulsifiers as determined by viscosity measurement. J. Food Sci..

[13] Lv Y., Zhang L., Li M., He X., Hao L., Dai Y. (2019). Physicochemical properties and digestibility of potato starch treated by ball milling with tea polyphenols. Int. J. Biol. Macromol..

[14] Hussain S., Mohamed A.A., Alamri M.S., Ibraheem M.A., Qasem A.A.A., Shazad S.A., Ababtain I.A. (2020). Use of gum cordia (*Cordia myxa*) as a natural starch modifier; effect on pasting, thermal, textural, and rheological properties of corn starch. Foods.

[15] Yan W., Zhang M., Zhang M., Yadav M.P., Jia X., Yin L. (2022). Effect of wheat bran arabinoxylan on the gelatinization and long-term retrogradation behavior of wheat starch. Carbohydr. Polym..

[16] Rojas J.A., Rossell C.M., Benedito de Barber C. (1999). Pasting properties of different wheat flour-hydrocolloid systems. Food Hydrocoll..

[17] Shi X., BeMiller J.N. (2002). Effects of food gums on viscosities of starch suspensions during pasting. Carbohydr. Polym..

[18] Qiu S., Yadav M.P., Liu Y., Chen H., Tatsumi E., Yin L. (2016). Effects of corn fiber gum with different molecular weights on the gelatinization behaviors of corn and wheat starch. Food Hydrocoll..

[19] Tester R.F. (1997). Influence of growth conditions on barley starch properties. Int. J. Biol. Macromol..

[20] Xie H., Ying R., Huang M. (2022). Effect of arabinoxylans with different molecular weights on the gelling properties of wheat starch. Int. J. Biol. Macromol..

[21] Funami T., Kataoka Y., Omoto T., Goto Y., Asai I., Nishinari K. (2005). Effects of non-ionic polysaccharides on the gelatinization and retrogradation behavior of wheat starch. Food Hydrocoll..

[22] Funami T., Kataoka Y., Noda S., Hiroe M., Ishihara S., Asai I., Takahashi R., Nishinari K. (2008). Functions of fenugreek gum with various molecular weights on the gelatinization and retrogradation behaviors of corn starch-1: Characterizations of fenugreek gum and investigations of corn starch/fenugreek gum composite system at a relatively high starch concentration; 15w/v%. Food Hydrocoll..

[23] Biliaderis C.G., Arvanitoyannis I., Izydorczyk M.S., Prokopowick D.J. (1997). Effect of Hydrocolloids on gelatinization and structure formation in concentrated waxy maize and wheat starch gels. Starch/Stärke.

[24] Richardson S.J., Baianu I.C., Steinberg M.P. (1987). Mobility of water in sucrose solutions determined by deuterium and oxygen-17 nuclear magnetic resonance measurements. J. Food Sci..

[25] Si X., Li T., Zhang Y., Zhang W., Qian H., Li Y., Zhang H., Qi X., Wang L. (2021). Interactions between gluten and water-unextractable arabinoxylan during the thermal treatment. Food Chem..

[26] Wang P., Zou M., Tian M., Gu Z., Yang R. (2018). The impact of heating on the unfolding and polymerization process of frozen-stored gluten. Food Hydrocoll..

[27] Rosell C.M., Foegeding A. (2007). Interaction of hydroxypropylmethylcellulose with gluten proteins: Small deformation properties during thermal treatment. Food Hydrocoll..

[28] Slade L., Levine H. (1991). Beyond water activity: Recent advances based on an alternative approach to the assessment of food quality and safety. Crit. Rev. Food Sci. Nutr..

[29] Morrison W.R., Tester R.F., Gidley M.J. (1994). Properties of damaged starch granules II. Crystallinity, molecular order and gelatinization of ball milled starches. J. Cereal Sci..

[30] Tester R.F. (1997). Properties of damaged starch granules: Composition and swelling properties of maize, rice, pea and potato starch fractions in water at various temperatures. Food Hydrocoll..

[31] Barrera G.N., León A.E., Ribotta P.D. (2012). Effect of damaged starch on starch thermal behavior. Starch/Stärke.

[32] Gudmundsson M., Eliasson A.C., Bengtsson S., Aman P. (1991). The effects of water soluble arabinoxylan on gelatinization and retrogradation of starch. Starch/Stärke.

[33] Kim W.W., Yoo B. (2011). Rheological and thermal effects of galactomannan addition to acorn starch paste. Food Sci. Technol..

[34] Biliaderis C.G., Maurice T.J., Vose J.R. (1980). Starch gelatinization phenomena studied by differential scanning calorimetry. J. Food Sci..

[35] Krüger A., Ferrero C., Zaritzky N.E. (2003). Modelling corn starch swelling in batch systems: Effect of sucrose and hydrocolloids. J. Food Eng..

[36] Ratnayake W.S., Jackson D.S. (2007). A new insight into the gelatinization process of native starches. Carbohydr. Polym..

[37] Biliaderis C.G., Page C.M., Maurice T.J., Juliano B.O. (1986). Thermal Characterization of rice starches: A polymeric approach to phase transitions of granular starch. J. Agric. Food Chem..

[38] BeMiller J.N. (2011). Pasting, paste, and gel properties of starch-hydrocolloid combinations. Carbohydr. Polym..

[39] Grossutti M., Dutcher J.R. (2016). Correlation between chain architecture and hydration water structure in polysaccharides. Biomacromolecules.

[40] Shahzad S.A., Hussain S., Mohamed A.A., Alamri M.S., Ibraheem M.A., Abdo Qasem A.A. (2019). Effect of hydrocolloid gums on the pasting, thermal, rheological and textural properties of chickpea starch. Foods.

[41] Varela M.S., Navarro A.S., Yamul D.K. (2016). Effect of hydrocolloids on the properties of wheat/potato starch mixtures. Starch Stärke.

[42] Lee M.H., Baek M.A., Cha D.S., Park H.J., Lim S.T. (2002). Freeze-thaw stabilization of sweet potato starch gel by polysaccharide gums. Food Hydrocoll..

