# Supplementary material for: Effect of Damaged Starch and Wheat-Bran Arabinoxylans on Wheat Starch and Wheat Starch–Gluten Systems
Source: Foods. 2024 Feb 24;13(5):689. doi: 10.3390/foods13050689 (PMC10930467; doi:10.3390/foods13050689)
Supplement: Supplementary file 1 [file foods-13-00689-s001.zip › foods-2872217-supplementary.pdf]

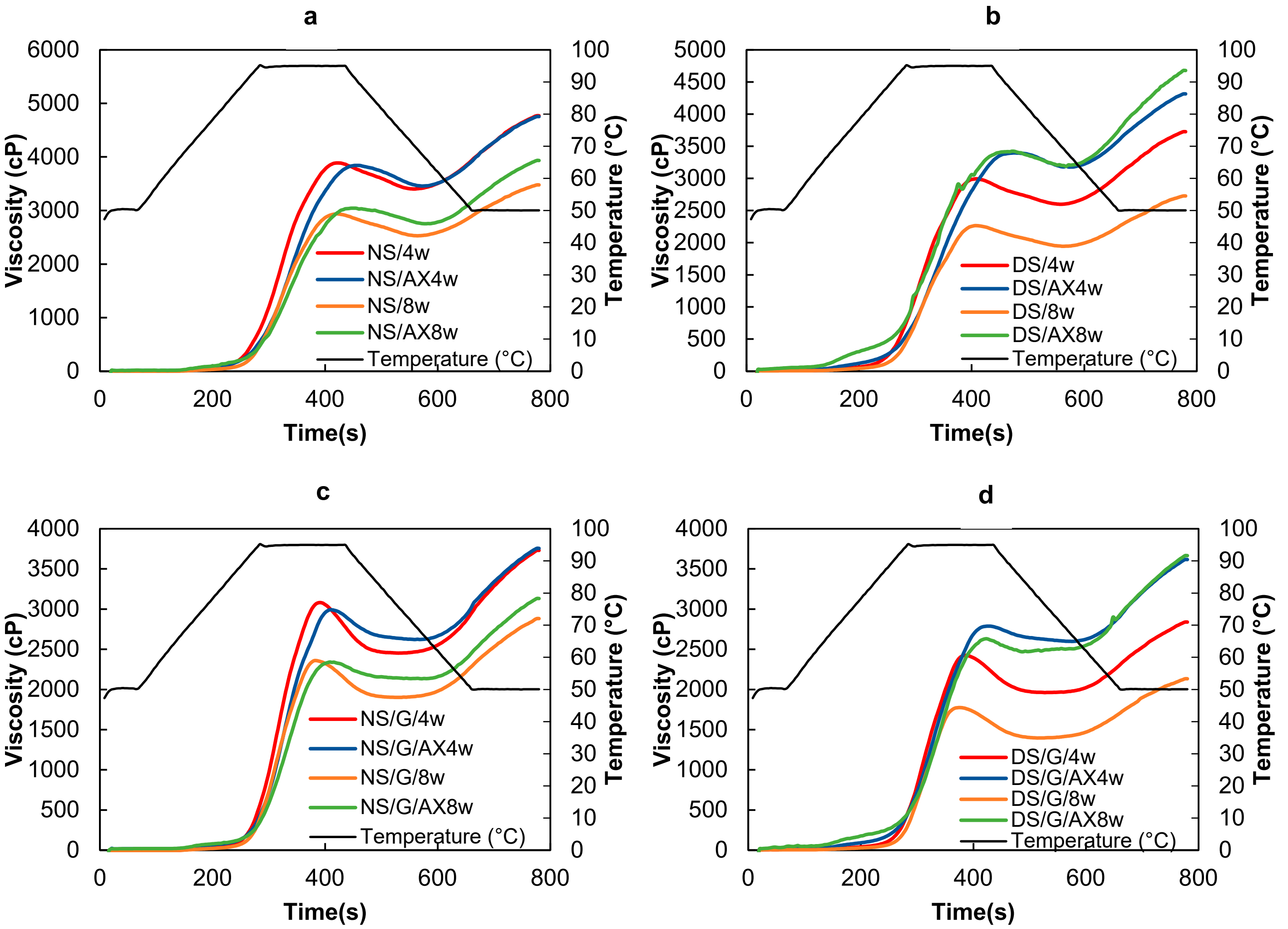

Figure S1.

Effect of arabinoxylans and damaged starch on the starch and starch-gluten pasting profiles in water. a) NS/4w and NS/AX4w: 11.9% w/w, NS/8w and NS/AX8w: 11.4% w/w, b) DS/4w and DS/AX4w: 11.9% w/w, DS/8w and DS/AX8w: 11.4% w/w, c) NS/G/4w and NS/G/AX4w: 10.8% w/w, NS/G/8w and NS/G/AX8w: 10.4% w/w, d) DS/G/4w and DS/G/AX4w: 10.8% w/w, DS/G/8w and DS/G/AX8w: 10.4% w/w.

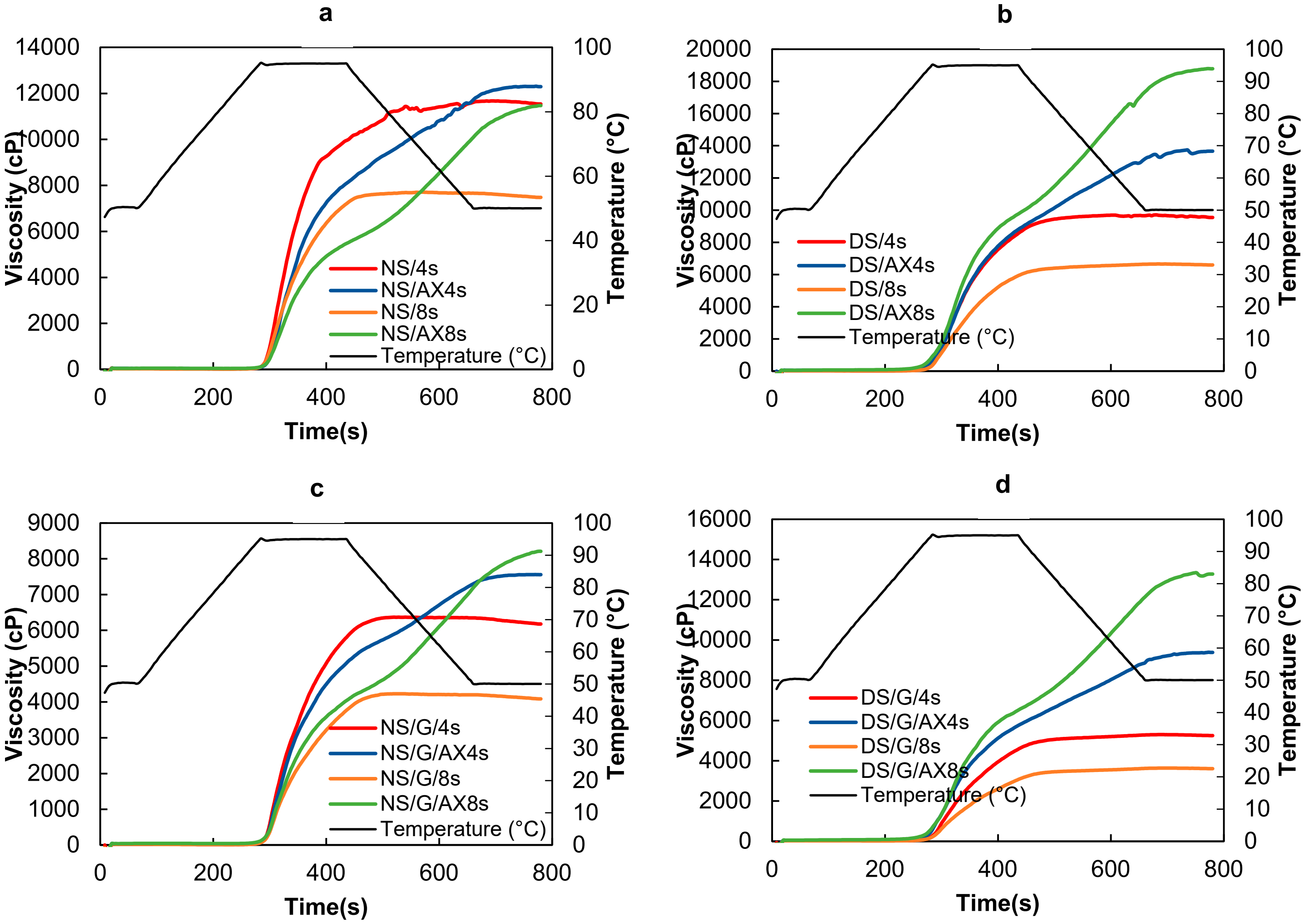

Figure S2.

Effect of arabinoxylans and damaged starch on the starch and starch-gluten pasting profiles in water. a) NS/4s and NS/AX4s: 11.9% w/w, NS/8s and NS/AX8s: 11.4% w/w, b) DS/4s and DS/AX4s: 11.9% w/w, DS/8s and DS/AX8s: 11.4% w/w, c) NS/G/4s and NS/G/AX4s: 10.8% w/w, NS/G/8s and NS/G/AX8s: 10.4% w/w, d) DS/G/4s and DS/G/AX4s: 10.8% w/w, DS/G/8s and DS/G/AX8s: 10.4% w/w.
